# Myoclonic status epilepticus and cerebellar hypoplasia associated with a novel variant in the *GRIA3* gene

**DOI:** 10.1007/s10048-021-00666-1

**Published:** 2021-11-03

**Authors:** Berardo Rinaldi, Yu-Han Ge, Elena Freri, Arianna Tucci, Tiziana Granata, Margherita Estienne, Jia-Hui Sun, Bénédicte Gérard, Allan Bayat, Stephanie Efthymiou, Cristina Gervasini, Yun Stone Shi, Henry Houlden, Paola Marchisio, Donatella Milani

**Affiliations:** 1 Fondazione IRCCS Ca’ Granda Ospedale Maggiore Policlinico, Milan, Italy; 2 Ministry of Education Key Laboratory of Model Animal for Disease Study, Department of Neurology, Drum Tower Hospital, Medical School, Nanjing University, Nanjing, China; 3 State Key Laboratory of Pharmaceutical Biotechnology, Model Animal Research Center, Institute for Brain Sciences, Chemistry and Biomedicine Innovation Center, Nanjing University, Nanjing, China; 4 Department of Pediatric Neuroscience, Fondazione IRCCS Istituto Neurologico C. Besta, Milan, Italy; 5 Clinical Pharmacology, William Harvey Research Institute, School of Medicine and Dentistry, Queen Mary University of London, London, EC1M 6BQ UK; 6 Laboratoires de diagnostic génétique, Institut Medical d’Alsace, Hôpitaux Universitaire de Strasbourg, Strasbourg, France; 7 Department for Genetics and Personalized Medicine, Danish Epilepsy Centre, Dianalund, Denmark; 8 Institute for Regional Health Services Research, University of Southern Denmark, Odense, Denmark; 9 Department of Neuromuscular disorders, UCL Queen Square Institute of Neurology, London, UK; 10 Medical Genetics, Department of Health Sciences, Università degli Studi di Milano, Milan, Italy

**Keywords:** *GRIA3*, AMPARs, Glutamate, Myoclonic status epilepticus, Cerebellar hypoplasia

## Abstract

AMPA-type glutamate receptors (AMPARs) are postsynaptic ionotropic receptors which mediate fast excitatory currents. AMPARs have a heterotetrameric structure, variably composed by the four subunits GluA1-4 which are encoded by genes *GRIA1*-*4*. Increasing evidence support the role of pathogenic variants in *GRIA1-4* genes as causative for syndromic intellectual disability (ID). We report an Italian pedigree where some male individuals share ID, seizures and facial dysmorphisms. The index subject was referred for severe ID, myoclonic seizures, cerebellar signs and short stature. Whole exome sequencing identified a novel variant in *GRIA3*, c.2360A > G, p.(Glu787Gly). The *GRIA3* gene maps to chromosome Xq25 and the c.2360A > G variant was transmitted by his healthy mother. Subsequent analysis in the family showed a segregation pattern compatible with the causative role of this variant, further supported by preliminary functional insights. We provide a detailed description of the clinical evolution of the index subjects and stress the relevance of myoclonic seizures and cerebellar syndrome as cardinal features of his presentation.

## Introduction

The *GRIA3* gene is located at Xq25 and encodes the AMPA-type glutamate receptor (AMPAR) subunit 3. AMPARs belong to ionotropic glutamate receptors and are responsible for fast excitatory postsynaptic currents. AMPARs are heterotetrameric receptors combining four different subunits, defined GluA1-4 and encoded by *GRIA1-4* genes, respectively. GluA1-4 are differentially expressed during brain development and across the central nervous system [1, 2]. AMPAR functional properties depend on the composition of the heterotetramer, their post-translational modifications and exposure rate onto the postsynaptic membrane [3–5]. Moreover, all the AMPAR genes express in vivo two different isoforms, generated by means of alternative splicing of exon 14 (flip/flop isoforms), which provides different kinetic behaviours and contributes to the functional richness of AMPARs [3, 6]. This tight regulation contributes to learning and memory processes via the synaptic plasticity [7–9].

Genetic variability in AMPAR genes has been associated with different neurological traits in humans such as sleep pattern [10], migraine [11, 12] and psychoactive drug addiction [13, 14]. Currently, pathogenic variants in all the AMPAR genes have been reported in subjects presenting with syndromic intellectual disability. Deleterious variants currently described in *GRIA1*, *GRIA2* and *GRIA4*, mapping respectively to chromosome 1, 4 and 11, are all de novo [15–17] whereas those in *GRIA3* are mostly inherited from presumably healthy carrier mothers. Specifically for *GRIA3*, six unrelated pedigrees have been reported so far [18–20] and recurrent clinical features include intellectual disability, behavioural disorders and epilepsy. The employment of new sequencing techniques has identified further individuals, ascertained for early-onset epileptic encephalopathy, neurodevelopmental disorder, movement disorders and ID associated with abnormal sleep pattern [20–25]. Besides single nucleotide variants, the causative role of *GRIA3* in XLID patients has been suggested also for Xq25 copy number variants [18, 26–30] (CNVs) and for a balanced t(X;12)(q24;q15) translocation disrupting the *GRIA3* gene [31].

In the present study, we present a five-generation Italian pedigree with a novel *GRIA3* variant co-segregating with a neurological phenotype in males.

## Methods

### WES

The proband has been evaluated with a long-term follow-up combining neurological and genetic expertise. The family expressed written consent for the publication. Genomic DNA was extracted from peripheral blood samples according to standard procedures of phenol chloroform extraction. WES on the trio was performed as described elsewhere [32] in Macrogen, Korea. Briefly, target enrichment was performed with 2 μg genomic DNA using the SureSelectXT Human All Exon Kit version 6 (Agilent Technologies, Santa Clara, CA, USA) to generate barcoded WES libraries. Libraries were sequenced on the HiSeqX platform (Illumina, San Diego, CA, USA) with 50 × coverage. Quality assessment of the sequence reads was performed by generating QC statistics with FastQC. Our bioinformatics filtering strategy included screening for only exonic and donor/acceptor splicing variants. In accordance with the pedigree and phenotype, priority was given to rare variants (< 0.01% in public databases, including 1000 Genomes project, NHLBI Exome Variant Server, Complete Genomics 69 and Exome Aggregation Consortium [ExAC v0.2]) that were fitting a dominant (heterozygous/hemizygous) or a de novo model and/or variants in genes previously linked to intellectual disability and other neurological disorders. Variants of interest were confirmed with Sanger sequencing. Amplification reactions were performed with standard FastStart PCR reagents (Roche), on an ABI Veriti Thermal Cycler (Applied Biosystems). PCR products were purified using Exo-SAP (Exonuclease I and Shrimp Alkaline Phosphatase) and sequencing PCR was performed bi-directionally using BigDye Terminator Ready Reaction Mix kit version 3.1 (Applied Biosystems) and analyzed on an ABI 3730xl capillary sequencer. Electropherograms were generated on the Sequencher software to compare sequences of probands versus healthy controls. A similar approach was applied to conduct the segregation analysis in parents and in two male relatives. The significance of the identified variant was classified according to American College of Medical Genetics and Genomics (ACMG) criteria using Varsome tool [33].

### cDNA constructs

cDNAs encoding human flop- and flip-type GluA3 (GluA3o and GluA3i, respectively) were subcloned into the NheI and XhoI restriction sites of the vector pCAGGS-IRES-EGFP. An HA epitope was inserted after the signal peptide (SP) of GluA3o, which read SP-GGGGS-HA-GGGGS-GluA3o. The GluA3o in this paper contains this HA epitope if not otherwise stated. Human GluA2 was subcloned into the vector pCAGGS-IRES-mCherry. Coexpression of GluA2 and GluA3 was identified by the fluorescence of EGFP and mCherry. GluA3i_E787G and GluA3o_E787G were made by overlapping PCR and confirmed by Sanger sequencing.

### HEK cells

HEK293T cells were cultured in a 37 °C incubator supplied with 5% CO2. Transfection was performed in 35-mm dishes using lipofectomine2000 reagents (Invitrogen). When coexpression was carried out, the ratio of GluA3 to GluA2 cDNA was 1:1. NBQX (100 µM) was included in culture media to block AMPAR-induced cytotoxicity. Cells were dissociated with 0.05% trypsin and plated on coverslips pretreated with poly-D-lysine 24-h post transfection. Recording was performed 4 h after plating.

### Electrophysiology

Receptor desensitization was recorded with whole-cell configuration of the transfected HEK 293 T cells. The external solution was as follows (in mM): 140 NaCl, 2.5 KCl, 2 CaCl2, 1 MgCl2, 5 glucose and 10 HEPES (pH 7.4). Patch pipettes (resistance 3 to 5 MΩ) were filled with a solution containing the following (in mM): 130 KF, 33 KOH, 2 MgCl2, 1 CaCl2, 11 EGTA and 10 HEPES (pH 7.4). Glutamate (10 mM) diluted into the external solution was applied to lifted HEK cells with whole-cell configuration for 500 ms with a theta glass pipette mounted on a piezoelectric bimorph every 5 s [34]. Glutamate-induced currents were recorded with holding potential of − 70 mV. All the currents were collected with an Axoclamp 700B amplifier and Digidata 1440A (MolecularDevices, Sunnyvale, CA, USA), filtered at 2 kHz and digitized at 100 kHz. The current data were analyzed using Clampfit software.

## Results

**Fig. 1:**
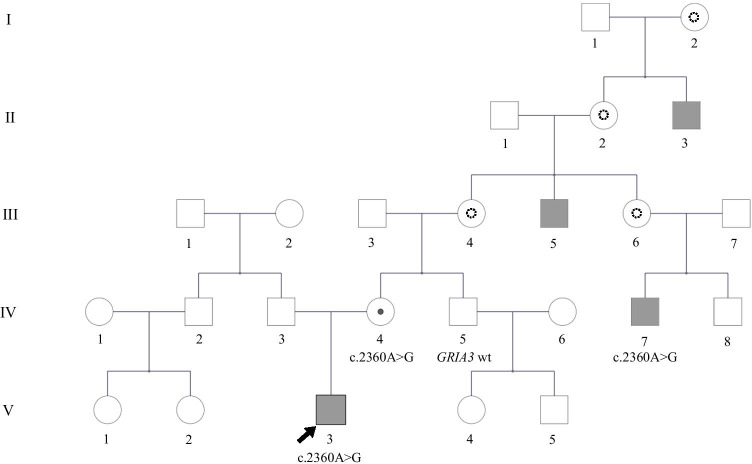
Pedigree of the index family. Symptomatic individuals are shown in grey; the black arrow identifies the index subject; dashed circles identify possible female carriers

The proband was referred at the age of 11 years for undiagnosed ID, drug-resistant epilepsy and ataxia. He was born to a healthy non consanguineous Italian couple. The family history revealed a 4th-degree relative with Down syndrome and four male relatives in the maternal lineage with variable association of ID, seizures and dysmorphisms (Fig. 1). The pregnancy was uneventful, except for few episodes of first-trimester bleeding. Chorionic villus sampling was performed due to the family history, showing a normal male karyotype. He was born at term and the neonatal weight was 2730gr. During the first month, he underwent surgery for pyloric stenosis.

**Fig. 2:**
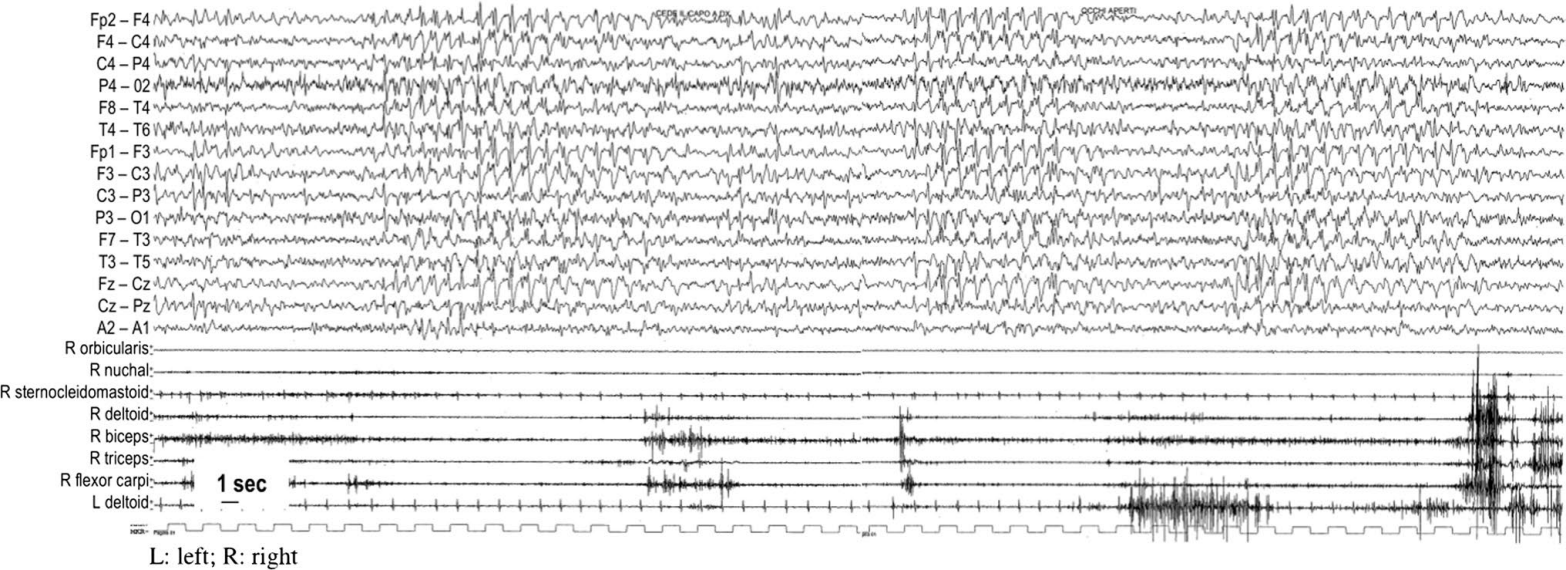
EEG and electromyographic polygraphy during myoclonic status epilepticus (recorded at the age of 5 years, amplitude: 400 microVolt/cm)

Psychomotor development was initially normal. Developmental delay and gait instability was firstly noticed at 15 months. Epilepsy onset occurred at 29 months with clonic seizures and myoclonic seizures with fall. Neurological examination was characterized by a cerebellar syndrome with ataxia, tremor and dysmetria. Interictal EEG revealed a poor organization of background activity, with bilateral high voltage activity and was dominated by epileptiform abnormalities prevalent on bilateral frontal regions; ictal discharges consisted of bilateral frontal or diffuse spike and wave. From the onset on, seizures quickly became very frequent and drug-resistant with recurrent myoclonic status epilepticus (Fig. 2). The patient was unsuccessfully treated with several therapeutic approaches: valproate, ethosuximide, methosuximide, clobazam, steroids such as ACTH and hydrocortisone, IGIV, bromide and clonazepam.

**Fig. 3:**
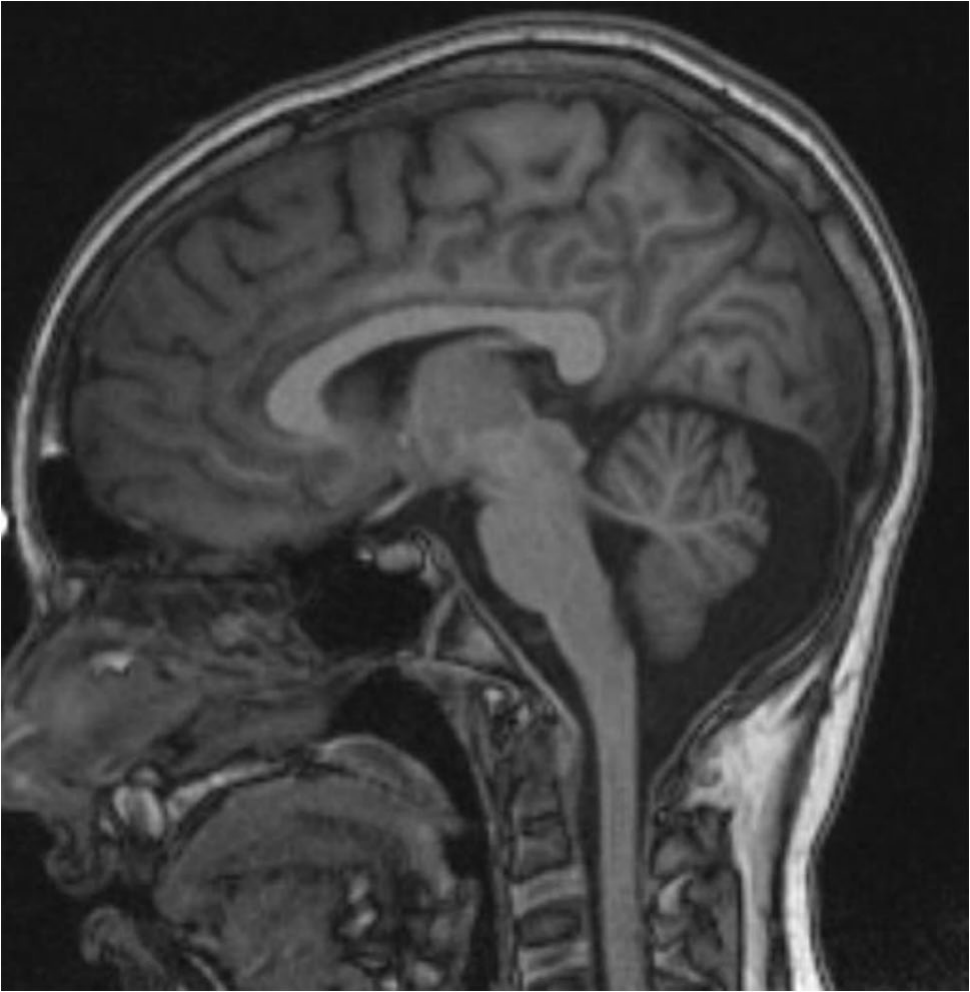
Sagittal T1-weighted cerebral MRI scan showing vermian hypoplasia

1,5-Tesla cerebral MRI described cerebellar vermis hypoplasia and a T1-weighted hyperintensity at the right frontal region, reviewed as a cortical dysplasia lesion by means of 3-Testla MRI (Fig. 3). Spectroscopy was normal.

**Fig. 4:**
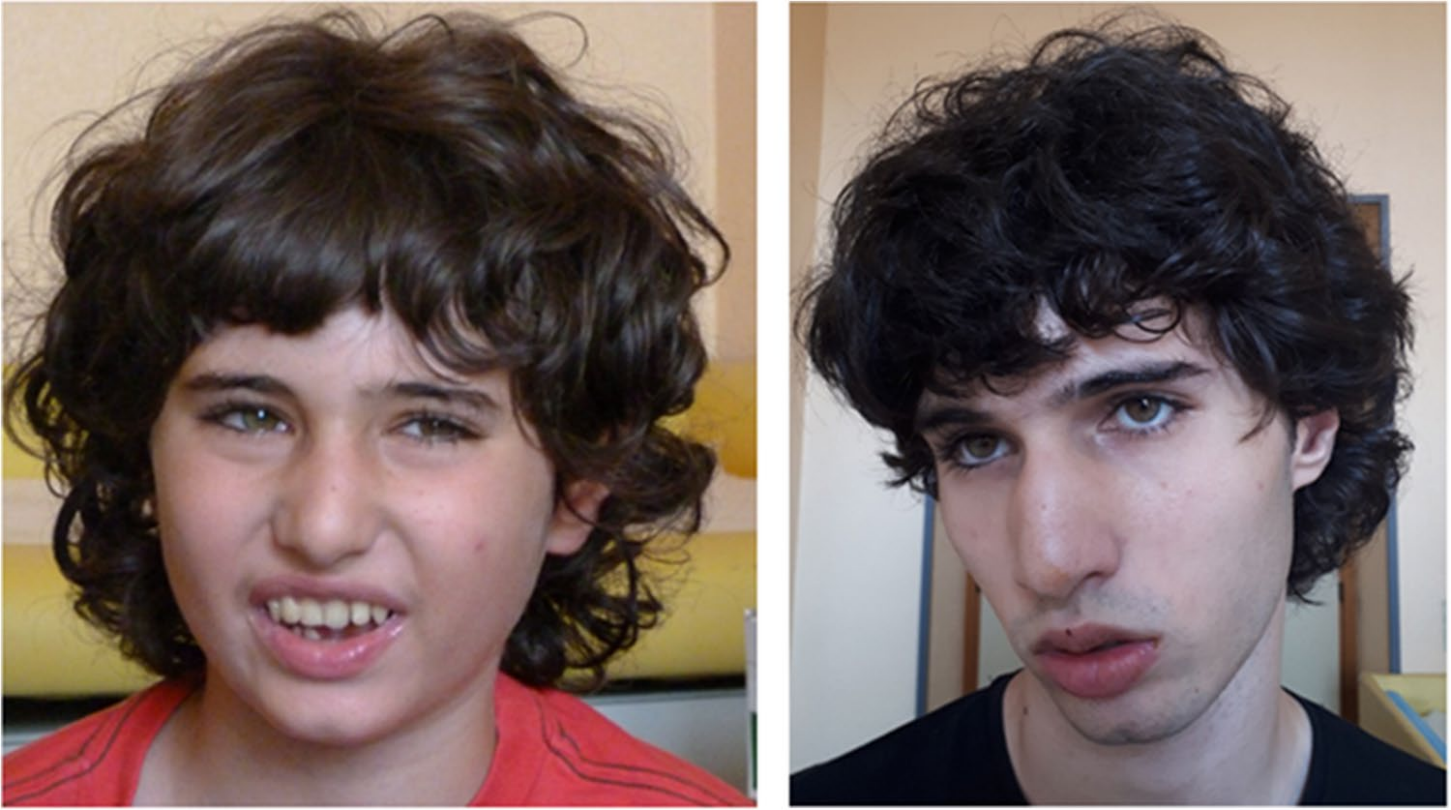
Facial appearance of the index subject at the age of 11 (left) and 18 (right)

His last physical evaluation was at the age of 18 years. Height was 161 cm (< 3° percentile), weight was 47.5 kg (< 3° percentile) and OFC was 57 cm (90° percentile). He showed horizontal eyebrow, long eyelashes, bulbous nasal tip, underfolded helix, short philtrum, everted lower lip, and mild retrognathia (Fig. 4). Neurological examination revealed ataxia, severe intellectual disability with poor language and dysarthria, and behavioural disorder consisting in aggressive outbursts. From the age of 14, he became seizure-free, being on a very low dosage of ethosuximide and clonazepam. His last EEG was performed at 17 years old and showed interictal diffuse epileptiform abnormalities during sleep.

**Fig. 5:**
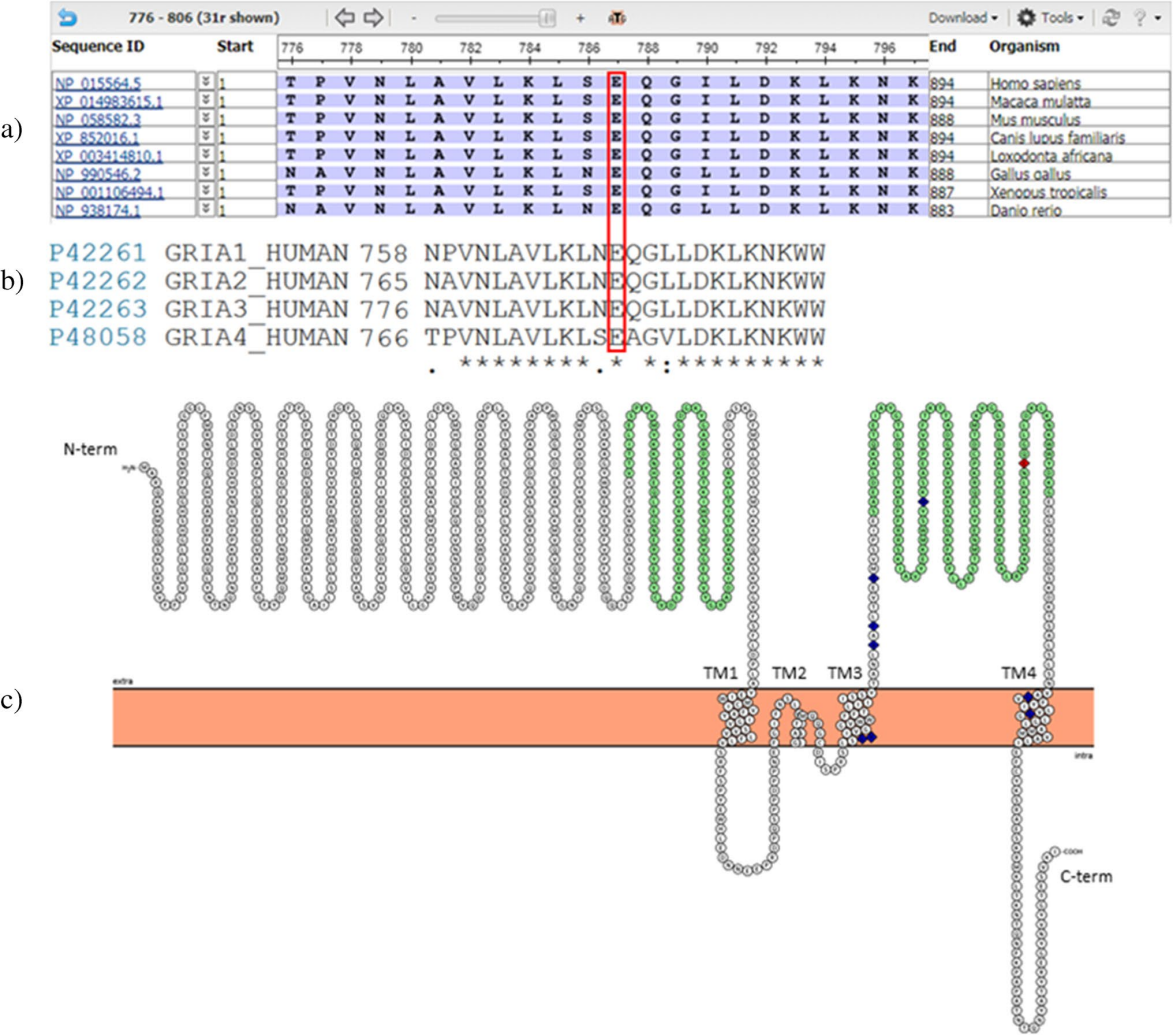
Conservation and localization of the Glu787 residue. (a) Phylogenetic conservation among different species; (b) conservation among the primary structure of human GluA1-4 subunits; (c) graphical representation of the GluA3 subunit, published mutated residues 18, 19, 20, 22, 23, 25 are shown in blue, the Glu787 residue in red. The green residues constitute the ligand-binding domain (LBD). Figure 5a, 5b and 5c were prepared with the help of COBALT (NCBI), UniProt and Protter, respectively (please refer to Web Resources)

In addition to 1st-tier metabolic screening, he underwent several genetic testing, all with normal results: *FMR1* trinucleotide expansion testing, chromosomal microarray (CMA), Sanger sequencing of *OPHN1* gene and targeted resequencing of *SLC9A6*, *ARX*, *MECP2*, *CDKL5*, *FOXG1*, *UBE3A* genes. WES identified two variants satisfying the filtering approach and no other class IV/V variants were found in his exome. The first one is the *SLC16A2* gene variant NC_000023.10:g.73744270G > A (NM_006517:c.652G > A, p.(Val218Ile)), which is present in Gnomad (AF 0.00001) and was interpreted as not causative also in view of the normality of the thyroid hormone profiles in the proband. The second one is the *GRIA3* gene variant NC_000023.10:g.122599560A > G (NM_000828:c.2360A > G, p.(Glu787Gly)), inherited from the asymptomatic mother. This variant maps to the exon 14 of the canonical transcript (NM_000828, flop isoform) whereas results in the intronic variant c.2324 + 597A > G in the alternative isoform (NM_007325.5, flip isoform). When considering the canonical transcript, this variant was classified as likely pathogenic (PM1, PM2, PP1, PP2, PP3, PP4). In particular, this variant is absent in the GnomAD database and is predicted as deleterious by several bioinformatic pathogenicity scores (CADD score: 29.8; MutationTaster: disease causing; Sift4G: Damaging; PolyPhen-2: probably damaging). The Glu787 residue is conserved among species and in the primary sequence of the other AMPAR subunits as well (Fig. 5A-C). We conducted familial segregation analysis in one affected and in one unaffected relative of the maternal lineage (IV.7 and IV.5, respectively); as expected IV.7 carried the *GRIA3* variant whereas IV.5 did not.

**Fig. 6:**
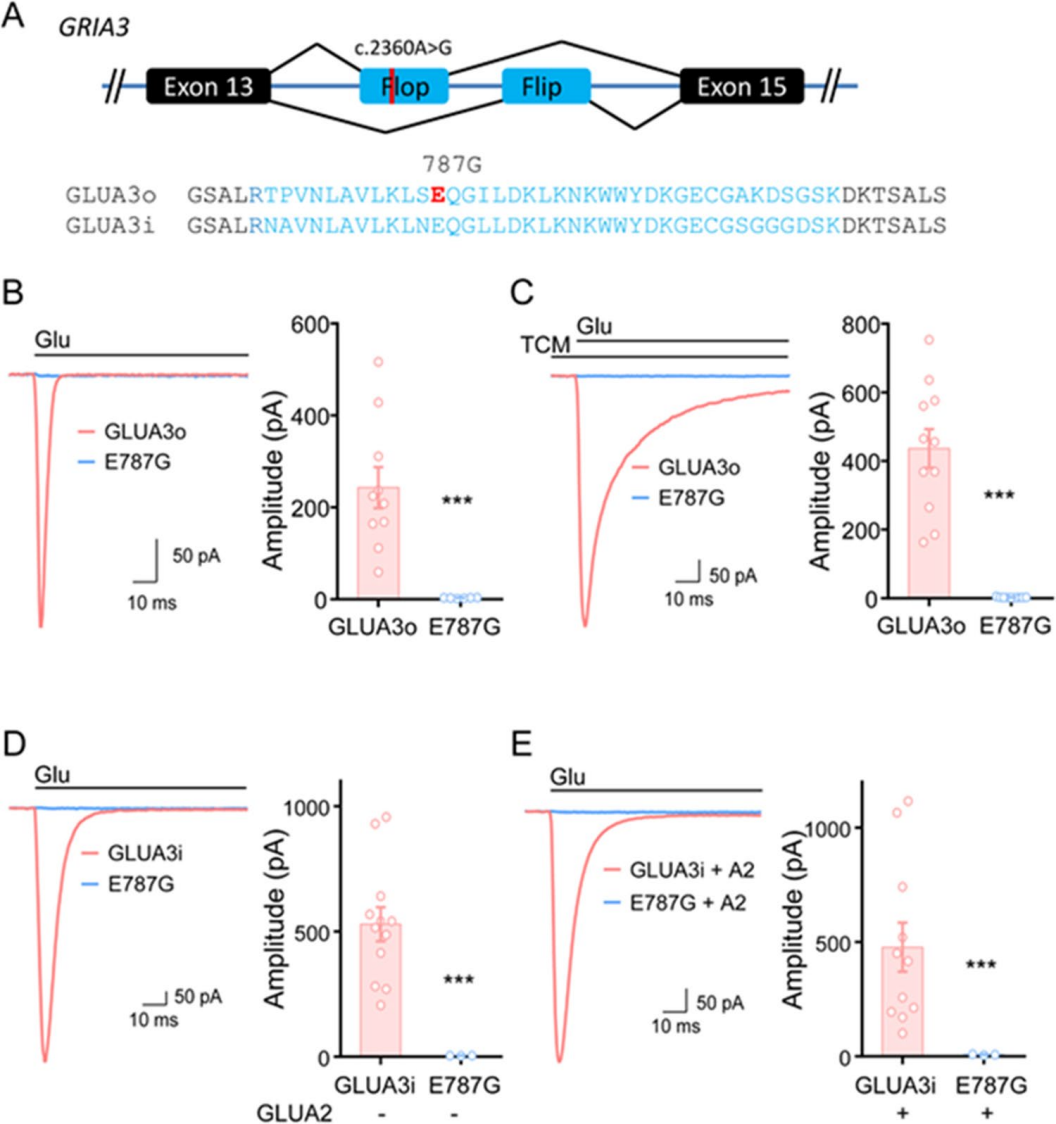
Glutamate-induced currents in wild-type GluA3 and in the E787G mutant. (A) The flip and flop of GLUA3. (B) Glutamate-induced currents in GLUA3o and GLUA3o_E787G. Left panel, sample traces. Right panel, bar graph showing the glutamate-induced current amplitudes. GLUA3o, 242.6 ± 44.5 pA, *n* = 10; GLUA3o_E787G, 2.9 ± 0.3 pA, *n* = 6. (C) Glutamate-induced currents in GLUA3o and GLUA3o_E787G in the presence of TMC. GLUA3o, 436.2 ± 56.7 pA, *n* = 11; GLUA3o_E787G, 2.6 ± 0.4 pA, *n* = 9. (D) Glutamate-induced currents in GLUA3i and GLUA3i-E787G. GLUA3i, 528.7 ± 67.7 pA, *n* = 12; GLUA3i_E787G, 3.6 ± 0.4, *n* = 3. (E) Glutamate-induced currents in GLUA3i and GLUA3i_E787G with the coexpression of GLUA2i. GLUA2/A3i, 477.1 ± 107.2 pA, *n* = 11; GLUA2/A3i_E787G, 7.8 ± 1.2, *n* = 3. Data are presented as means ± SEM. ***p < 0.001, unpaired *t*-test

To better understand whether the variant induces biological changes, we tested its effect on glutamate-induced current. We cloned the human GluA3 subunit and tested its function by expression in HEK cells. Since the variant is located in exon 14, we thus cloned both the flop- and flip-GluA3 (Fig. 6A, GLUA3o and GLUA3i, respectively). The two amino acid sequences differ in nine residues, albeit glutamic acid 787 is present in both. When glutamate (10 mM) was applied to HEK cells transfected with GluA3o and GluA3o_E787G, we observed the latter to produce no currents (Fig. 6B). In order to exclude that the E787G mutant desensitizes too fast to be recorded, we applied the AMPAR desensitization blocker, trichloromethiazide (TCM, 100 µM), together with glutamate and still measured no currents (Fig. 6C). Also in the case of the flip isoform, no glutamate-induced currents were recorded in GluA3i-E787G transfected HEK cells, whilst GluA3i induced significant currents (Fig. 6D). Since GluA3 forms heteromeric Ca2 + -impermeable AMPARs with GluA2 in the brain, we thus coexpressed GluA3i with GluA2i(R) and again we did not observe significant current in GluA2/A3i_E787G (Fig. 6E). Taken together these data support the pathogenic role of the E787G substitution and the functional importance of the variant g.122599560A > G in both the flip and flop isoform.

## Discussion

Neurodevelopmental disorders (NDD) and epilepsy often coexist in individuals referred for genetic investigations. More than distinct comorbidities, this reflects the variable clinical outcome of mutated genes with critical roles in the central nervous system [35]. As an example, ion channel dysfunction has been associated with a wide range of neurological conditions, either isolated or syndromic. Besides epilepsy and epileptic encephalopathies, the clinical spectrum of channelopathies also includes ataxia, movement disorders, ID, autism spectrum disorders (ASD), migraine, sleep disorders, cardiac arrhythmias and sudden unexpected death in epilepsy (SUDEP) [36]. In this context, few recent studies identified causative variants in genes encoding AMPAR subunits for individuals with NDD associated with epilepsy [15–17, 19, 24, 25].

Here, we report a novel *GRIA3* variant, c.2360A > G p.(Glu787Gly), co-segregating with a syndromic NDD in an Italian pedigree. The neurological phenotype of our proband mostly concerns three domains: developmental delay (DD) evolving in severe ID, cerebellar signs due to vermian hypoplasia and clonic/myoclonic seizures even configuring myoclonic status epilepticus. Moreover, he showed short stature, low weight, relative macrocephaly and facial dysmorphisms. This clinical presentation recalls what is already known for pathogenic variants in *GRIA3*, i.e. moderate-severe ID, seizures and myoclonic jerks, short stature with macrocephaly [18, 19, 24]. We show that the epileptic phenotype associated to *GRIA3* haploinsufficiency may manifest with refractory myoclonic status epilepticus, though the proband demonstrated an overall ameliorative evolution, becoming seizure-free from the age of 14. As to autism spectrum disorders and behavioural issues, also described in individuals with *GRIA3* deleterious variants, our patient did not express autistic traits whereas he occasionally showed oppositional conduct and aggressive outbursts. Cerebral MRI of our proband highlighted cerebellar vermis hypoplasia and a focal cortical dysplasia. Though robust neuroimaging data on male individuals with impairment of *GRIA3* have not been gathered yet, two patients with Xq25 duplications involving *GRIA3* revealed a thin corpus callosum and moderate superior vermian atrophy [26, 30]. On the other hand, their duplication includes other dosage-sensitive gene as *STAG2*, whose involvement may lead to corpus callosum abnormalities as well. With specific regard to the abnormal sleep pattern previously described in association with the p.(Ala653Thr) variant in *GRIA3* [22], any alteration of the circadian cycle was reported for our proband. Comparing the facial gestalt of our proband with others already published, few traits look recurrent, as for the hypotonic facies, malar flattening, short philtrum, thick vermillion of the lips with eversion of the lower lip [19, 30].

The identified p.(Glu787Gly) variant is located within the ligand-binding domain (LBD) of the receptor where glutamate binds and triggers the channel opening. Testing the functional effects of missense variants on synaptic receptors is puzzling as the amino acid change may lead to different and possibly discordant results depending on the state of the channel (active, inactive, desensitized). In our case, the scenario is further complicated by the location of the variant, being unknown a priori whether the variant is expressed (flop isoform) and to which extent.

Therefore, in this study, we started to characterize the *GRIA3* variant c.2360A > G p.(Glu787Gly). The absence of current of the mutant receptor in any of the tested settings may suggest that the g.122599560A > G variant leads to a loss of function of the subunit in both isoforms. If this result may be conceivable for the flop isoform, the mechanisms responsible for the impairment of the flip isoform remain to be determined. Further studies are then needed to expand our findings and provide a deeper understanding. A different pathogenic variant affecting the same amino acid residue was recently described in another Italian male child [24]. Interestingly, the two patients experienced status epilepticus and myoclonic seizures but with differences in epileptic severity and neuroimaging findings. Moreover, for the male patient reported by Trivisano et al., a targeted trial with a selective non-competitive AMPA receptor antagonist (Perampanel) proved to be ineffective, as expected in case of loss of function of the receptor (Fig. 6).

Because of the heterotetrameric structure of AMPARs, pathogenic variants in each of *GRIA1-4* genes might exert dominant effects on other subunits of AMPARs with convergent effects on the resulting phenotypes, as also suggested by functional studies [22]. Our proband shares some of the clinical features already associated with other *GRIA* genes. This includes early normal psychomotor development, speech delay more pronounced than the motor impairment, cerebellar MRI findings and neurological signs. Based on published data, the most overlapping pattern would appear with cases with pathogenic variants in *GRIA2* and, indeed, GluA3 co-assembles mostly with GluA2 to generate AMPARs [37]. On the other hand, the corresponding residue in the GluA2 subunit has already been found mutated (p.(Glu776Asp)) in an individual with a slightly different phenotype, consisting of DD/ID, ASD, insomnia, normal brain MRI and a single episode of generalized tonic seizure [16]. Therefore, the small numbers of patients and the wide range of functional effects produced by missense variants make stringent genotype–phenotype correlation for *GRIA* genes still uncertain.

## Web resources

CADD [38] (https://cadd.gs.washington.edu/)

COBALT (https://www.ncbi.nlm.nih.gov/tools/cobalt/re_cobalt.cgi)

FastQC (https://www.bioinformatics.babraham.ac.uk/projects/fastqc/)

GnomAD [39] (https://gnomad.broadinstitute.org/)

MutationTaster [40] (http://www.mutationtaster.org/)

PolyPhen-2 [41] (http://genetics.bwh.harvard.edu/pph2/)

Protter [42] (http://wlab.ethz.ch/protter/)

Sift4G [43] (https://sift.bii.a-star.edu.sg/sift4g/)

UniProt [44] (https://www.uniprot.org/)

VarSome [33] (https://varsome.com/)
