# Correction to: Myoclonic status epilepticus and cerebellar hypoplasia associated with a novel variant in the *GRIA3* gene

**DOI:** 10.1007/s10048-021-00678-x

**Published:** 2021-11-27

**Authors:** Berardo Rinaldi, Yu‑Han Ge, Elena Freri, Arianna Tucci, Tiziana Granata, Margherita Estienne, Jia‑Hui Sun, Bénédicte Gérard, Allan Bayat, Stephanie Efthymiou, Cristina Gervasini, Yun Stone Shi, Henry Houlden, Paola Marchisio, Donatella Milani

**Affiliations:** 1 Fondazione IRCCS Ca’ Granda Ospedale Maggiore Policlinico, Milan, Italy; 2 Ministry of Education Key Laboratory of Model Animal for Disease Study, Department of Neurology, Drum Tower Hospital, Medical School, Nanjing University, Nanjing, China; 3 State Key Laboratory of Pharmaceutical Biotechnology, Model Animal Research Center, Institute for Brain Sciences, Chemistry and Biomedicine Innovation Center, Nanjing University, Nanjing, China; 4 Department of Pediatric Neuroscience, Fondazione IRCCS Istituto Neurologico C. Besta, Milan, Italy; 5 Clinical Pharmacology, William Harvey Research Institute, School of Medicine and Dentistry, Queen Mary University of London, London, EC1M 6BQ UK; 6 Laboratoires de diagnostic génétique, Institut Medical d’Alsace, Hôpitaux Universitaire de Strasbourg, Strasbourg, France; 7 Department for Genetics and Personalized Medicine, Danish Epilepsy Centre, Dianalund, Denmark; 8 Institute for Regional Health Services Research, University of Southern Denmark, Odense, Denmark; 9 Department of Neuromuscular disorders, UCL Queen Square Institute of Neurology, London, UK; 10 Medical Genetics, Department of Health Sciences, Università degli Studi di Milano, Milan, Italy

**Correction to: Neurogenetics**

**https://doi.org/10.1007/s10048-021-00666-1**

In the original published article, the name of the seventh author has been misspelled.

“Jia-Hun Sun” should be “Jia-Hui Sun”.

This is being corrected in this publication.

